# Beyond the bite: understanding and managing post-arboviral pain

**DOI:** 10.1016/j.bjane.2024.844526

**Published:** 2024-06-22

**Authors:** Durval Campos Kraychete, Vinicius Borges Alencar, Eduardo Silva Reis Barreto, César Romero Antunes Júnior, Liliane Elze Falcão Lins-Kusterer, Guilherme Antonio Moreira de Barros, André P. Schmidt

**Affiliations:** a Universidade Federal da Bahia (UFBA), Faculdade de Medicina da Bahia (FMB), Salvador, BA, Brazil; b Universidade Estadual Paulista (UNESP), Faculdade de Medicina de Botucatu (FMB), Botucatu, SP, Brazil; c Hospital de Clínicas de Porto Alegre (HCPA), Serviço de Anestesia e Medicina Perioperatória, Porto Alegre, RS, Brazil; d Santa Casa de Porto Alegre, Serviço de Anestesia, Porto Alegre, RS, Brazil; e Hospital Nossa Senhora da Conceição, Serviço de Anestesia, Porto Alegre, RS, Brazil; f Universidade Federal do Rio Grande do Sul (UFRGS), Programa de Pós-graduação em Ciências Pneumológicas, Porto Alegre, RS, Brazil; g Universidade Federal do Rio Grande do Sul (UFRGS), Programa de Pós-graduação em Ciências Cirúrgicas, Porto Alegre, RS, Brazil; h Faculdade de Medicina da Universidade de São Paulo (FMUSP), Programa de Pós-Graduação em Anestesiologia, Ciências Cirúrgicas e Medicina Perioperatória, São Paulo, SP, Brazil

Arboviruses, a group of viral diseases transmitted by arthropods such as mosquitoes and ticks, have emerged as a growing global public health concern, especially as a consequence of the recently observed climate changes.^1^^,^^2^ In Brazil, the main arboviruses include Dengue, Zika, Yellow Fever, and Chikungunya. Viruses from the Togaviridae and Flaviviridae families are primarily associated with these diseases. Notably, the genus Alphavirus, particularly the Chikungunya virus, is recognized for its high arthritogenic potential.^1^^,^^3^ The viral cycle involves transmission by mosquitoes such as Aedes and Culex, and vertebrate hosts like monkeys and birds, which act as viral reservoirs.^1^

Post-arboviral pain is characterized by symptoms such as arthralgia, myalgia, and arthritis, which can persist for months or even years after the initial infection, significantly impacting patients' quality of life.^4^ A detailed understanding of the underlying pathophysiological mechanisms of this condition is crucial for developing effective management and treatment strategies, representing a promising area of investigation in medical research.

## Epidemiology

These diseases have a wide geographic distribution and a diverse evolutionary history, but climate changes may have impacted it.^2^ Dengue is a major arboviral disease with significant epidemiological importance, endemic in tropical and subtropical regions worldwide, including Brazil.^5^ Transmitted primarily by the *Aedes aegypti* mosquito, Dengue has seen a dramatic increase in the number of cases in recent decades in Brazil, especially in the years 2023 and 2024, with frequent endemic outbreaks, posing a major challenge to the Brazilian public health system.^1^^,^^5^ The Zika virus, first identified in Uganda in 1947, gained international attention during the 2015-2016 outbreak in Brazil. This outbreak revealed the potential for severe complications, such as microcephaly in newborns among other congenital abnormalities, collectively referred to as Congenital Zika Syndrome.^5^

Another notable arbovirus is Chikungunya, known for causing chronic arthralgia. This disease, originating in Tanzania, has spread to various regions globally. The name Chikungunya, derived from the Makonde dialect in Tanzania, means “that which bends up”, reflecting the severe joint pain and posture changes associated with the disease.^3^ Since the early 2000s, Chikungunya has caused significant outbreaks in Asia, the Pacific Ocean region, and more recently, Europe. Latin America, particularly Brazil, has experienced a substantial rise in Chikungunya cases since 2014, making it a high-incidence area.^5^ The spread of these viruses, often through urban vectors such as *Aedes aegypti*, represents a significant threat to public health globally. Effective vector control and public health strategies are critical in managing the impact of these diseases.^3^^,^^5^

## Pathophysiology

The pathophysiology of post-arboviral pain is a complex interaction involving various factors that influence disease progression, including viral and host-related aspects, and perpetuation of inflammatory signaling and cellular activation. The most studied chronic pathology is caused by the Chikungunya virus (CHIKV).^4^

Studies on the immunopathogenesis of arboviral infections often derive from animal models and in vitro studies.^6^ However, translating these findings into human immunity is not always straightforward, and viral replication can differ between humans and the primary animal models studied.^3^ Disease presentation varies between human and animal models, especially in the chronic phase.^6^

During the acute phase, arboviruses are usually inoculated into human skin through the bite of the mosquito vector. Salivary proteins from the vector modulate the local response, facilitating infection.^7^ After viral inoculation, immune cells near the site of inoculation are invaded by viral particles, initiating replication. This phase is characterized by rapid viral proliferation, coinciding with fever, skin rash, intense arthralgia, and leukopenia.^1^^,^^3^

**Figure 1:**
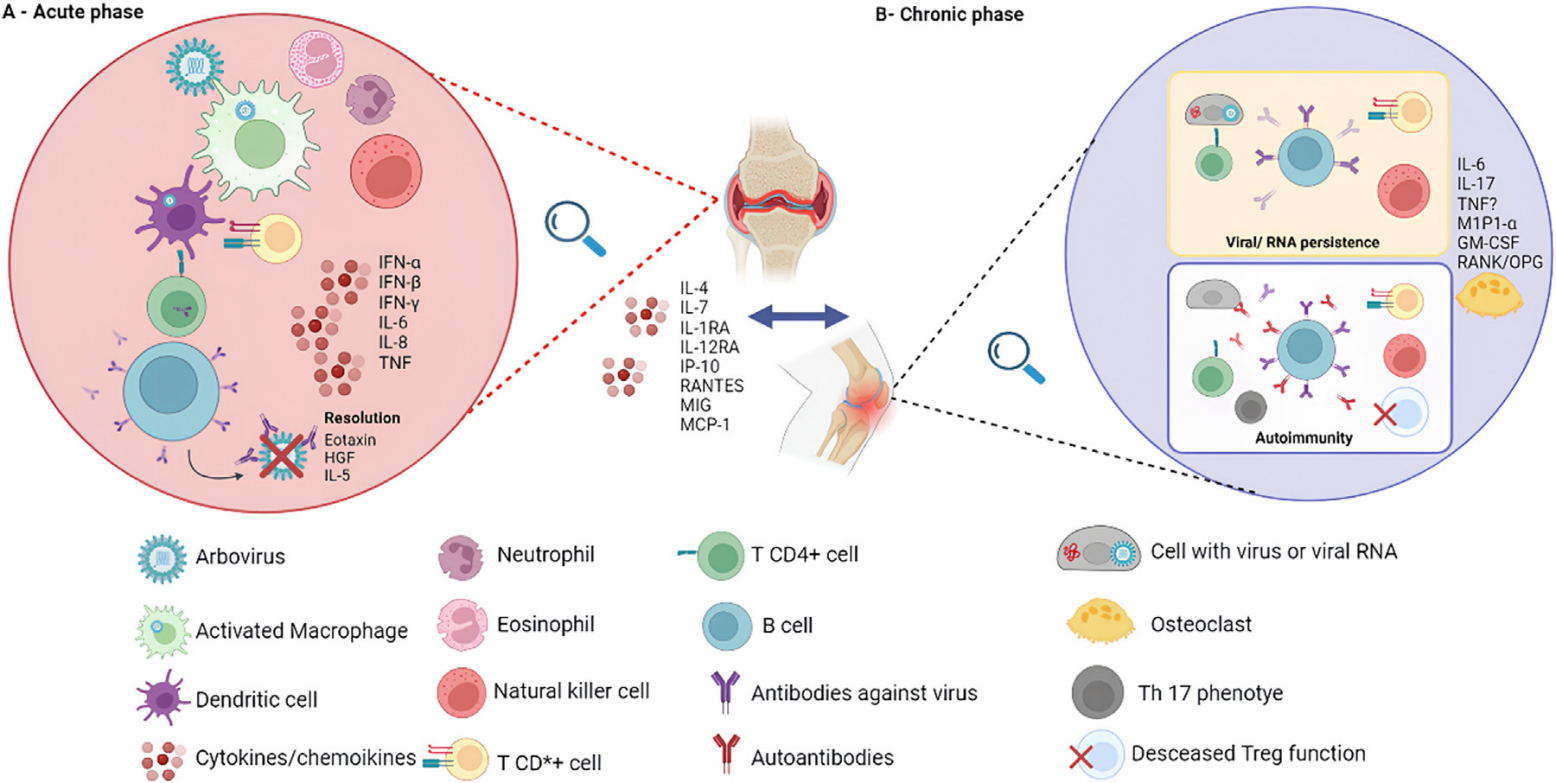
Simplified scheme of cellular, humoral, and soluble factors involved in the acute and chronic phases of Chikungunya fever In the acute phase (A), Chikungunya virus infection triggers an intense immune response, characterized by the activation of macrophages and monocytes, along with the massive production of pro-inflammatory cytokines and antibodies. This process is crucial for controlling initial viral replication but is also associated with acute symptoms, such as high fever and severe joint pain. In the chronic phase (B), which can persist for months to years after the initial infection, some hypotheses suggest that residual viral RNA fragments or proteins remain in macrophages and monocytes. This persistence can lead to continuous cellular activation, resulting in sustained production of inflammatory cytokines. Additionally, there is the possibility of the development of autoimmunity in genetically predisposed individuals, where the immune system attacks the body's own healthy tissues, contributing to chronic pain and inflammation. These mechanisms may explain the chronicity of musculoskeletal symptoms observed in many patients, such as persistent arthralgia and myalgia. All images in this figure were created using Biorender® (available in https://www.biorender.com) by Vinicius Alencar using Academic License Terms.

During the innate immune response, viral particles are primarily detected by mononuclear cells, triggering the production of various cytokines and chemokines, including type I interferon (IFN-α/β) response and subsequent production of IFN-γ and other pro-inflammatory cytokines, such as IL-6 and TNF-α.^8^ The adaptive immune response is initiated shortly after infection onset, with the activation of T and B lymphocytes. Antibody formation occurs early, with the production of IgM antibodies, followed by IgG.^8^ The persistence of chronic joint symptoms after the acute phase is not fully understood in Arboviruses but it is possibly related to failures in the control mechanisms of the initial inflammatory response.^1^ A simplified cellular scheme of the factors is available in Figure 1.

Studies in animal models and human patients have shown that CHIKV can persist in target tissues, such as joints, even after its elimination from the bloodstream.^7^^,^^9^ This suggests that the continuous presence of the virus in tissues may trigger a chronic inflammatory response, contributing to persistent pain and progression to chronic arthritic syndrome.^7^^,^^9^^,^^10^ However, the chronification of post-arboviral pain is still not fully understood, although evidence suggests it may be related to the persistence of viral RNA fragments or proteins in macrophages and monocytes, leading to cellular activation and subsequent cytokine and antibody production, as well as the induction of autoimmunity in genetically predisposed individuals.^9^^,^^10^ Prolonged immune cell activation and induction of autoimmunity in predisposed individuals may also contribute to symptom persistence.^10^

## Clinical manifestations

**Figure 2:**
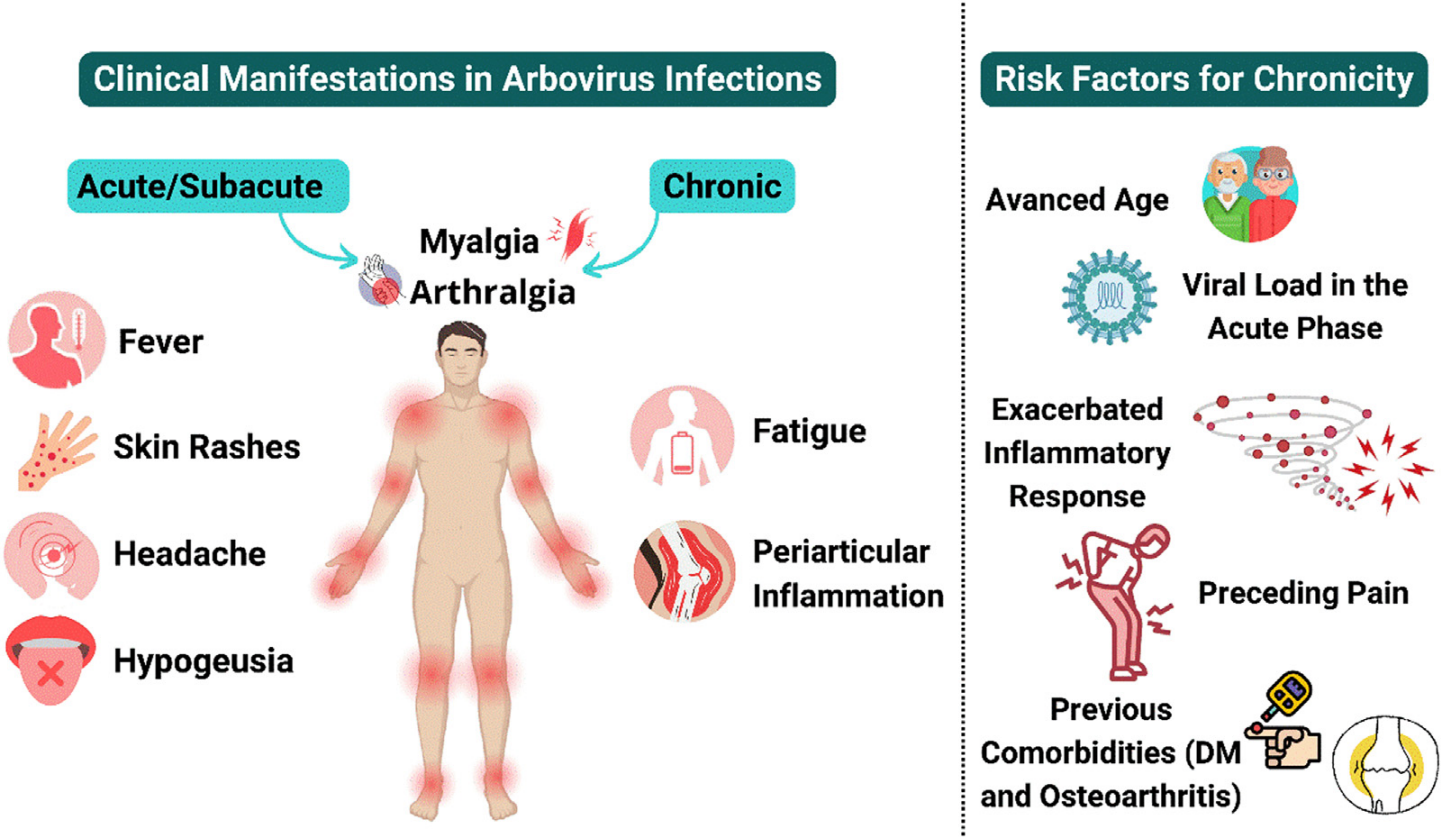
Clinical manifestations and risk factors in arbovirus infection Illustration of the main symptoms observed during the acute/subacute and chronic phases of arbovirus infections. In the acute/subacute phase, symptoms include fever, arthralgia (hands, ankles, and fingers), myalgia, skin rashes, erythroviolaceous macules, headache, and hypogeusia. In the chronic phase, predominant symptoms are persistent arthralgia in the same joints, myalgia, fatigue, and periarticular inflammation. The figure highlights the main risk factors for symptom chronicity: advanced age, high viral load during the acute phase, exacerbated inflammatory response, preceding pain, and pre-existing comorbidities such as diabetes mellitus (DM) and osteoarthritis. All images in this figure were created using Canva® (available in https://www.canva.com) by Vinicius Alencar using free license.

The symptoms and clinical signs following arboviral infections can vary and may persist for weeks, months, or even years after the initial infection (Fig. 2).^3^^,^^6^ During the acute phase of arboviral infection, symptoms may include fever, arthralgia, myalgia, and skin rashes, with variations in presentation depending on the patient and the epidemiological context.^2^ In some cases, the infection may be asymptomatic, while in others, symptoms can be severe and debilitating.^6^ While Chikungunya fever is widely studied, other common arboviruses, such as Dengue and Zika, lack similar investigations into their progression to the chronic phase.^1^^,^^4^

Chikungunya fever presents in three distinct phases: acute, subacute, and chronic. In the acute phase, symptoms may include fever, arthralgia, myalgia, and skin rashes. Some patients may be asymptomatic, while others exhibit severe and debilitating symptoms.^6^ After the acute phase, many patients continue to experience arthralgia and myalgia, which can persist even after other symptoms resolve. In a significant proportion of patients, these symptoms can evolve into a chronic arthritic syndrome, characterized by fluctuating joint pain primarily affecting extremities such as hands, ankles, and fingers. This pain is typically symmetrical and involves large and small appendicular joints.^6^^,^^11^

This chronic pain can have a significant impact on patients' quality of life, limiting daily activities and causing continuous discomfort.^12^ The transition to the chronic phase of Chikungunya fever can vary among patients, with some experiencing temporary remission followed by recurrence of joint symptoms. Studies indicate that between 25% and 50% of cases progress to the chronic phase, with a more pronounced tendency in patients infected with the East Central South African (ECSA) genotype of the virus.^12^^,^^13^ In this phase, clinical presentation can range from inflammatory joint involvement to diffuse non-inflammatory musculoskeletal manifestations, with some patients developing a fibromyalgia-like syndrome.^3^

A study conducted in Roraima, Brazil, investigated the impact of chronic arthritis caused by the Chikungunya virus ECSA genotype compared to local controls and patients with rheumatoid arthritis. The results revealed that, more than 2 years after infection, patients with CHIKV-induced arthritis reported moderate disease severity comparable to rheumatoid arthritis, with a greater impact on quality of life due to pain.^13^

Factors such as advanced age, high viral load during the acute phase of infection, and an exacerbated inflammatory response have been associated with a higher risk of developing chronic pain following arboviral infections.^7^ The specific mechanisms by which the virus persists in tissues and triggers a chronic inflammatory response are not yet fully understood and require further investigation.^4^^,^^9^

## Treatment

The available data on specific therapies for arboviral diseases at various stages are limited, reflecting the complexity of treatment strategies given the varied clinical evolution observed in these infectious scenarios.^14^ The lack of high-quality randomized clinical trials evaluating the efficacy of different treatments, along with methodological limitations in prospective studies, has posed significant challenges in defining robust therapeutic protocols.^11^^,^^14^

The therapeutic approach to Chikungunya fever, in particular, varies according to the phase of the disease and the pattern of joint manifestation.^14^ During the acute phase, the treatment aims to relieve pain using analgesics and opioids, emphasizing the importance of hydration, ice compresses for joint pain, and alternating rest with exercises to prevent joint dysfunction. In cases of pain with neuropathic characteristics, tricyclic antidepressants and anticonvulsants may be used.^3^^,^^6^

Corticosteroids are contraindicated during the acute phase due to the risk of increased viremia and recurrence of joint manifestations upon withdrawal. Non-steroidal anti-inflammatory drugs (NSAIDs) are also not recommended in the initial phase of infection due to the increased risk of hemorrhagic complications and are more appropriate once Dengue has been excluded, and when used after the seventh day.^3^^,^^7^ Although there is no approved antiviral treatment, some drugs have been reported to inhibit CHIKV replication, potentially reducing the chance of chronicity. These include favipiravir (T-705), MADTP-372, and arbidol.^3^^,^^7^^,^^14^

In the post-acute phase of Chikungunya, almost 50% of adult patients may experience persistent symptoms lasting up to 3 months.^13^ During this stage, although fever subsides, musculoskeletal complaints persist, characterized by polyarthralgia and/or polyarthritis of varying intensity, often affecting previously involved joints.^6^^,^^12^ Post-acute tenosynovitis, particularly in wrists and ankles, can emerge, along with morning stiffness. General symptoms such as asthenia, depression, and even alopecia may also be present.^9^

Treatment in this phase is similar to that in the acute phase, involving analgesics, opioids, anticonvulsants, and antidepressants as per the predominant symptoms. Physical therapy and rehabilitation play a crucial role in restoring joint mobility. NSAIDs have shown efficacy in 89% of patients and should be used for a period of 7 to 10 days, with possible extension if well-tolerated and effective, followed by gradual withdrawal.^3^ Corticosteroids may be considered after the failure of analgesics and NSAIDs, with recommended doses of prednisone ranging from 10 mg/day for mild to moderate cases to 0.5 mg.kg^−1^.day^−1^ for more severe cases.^11^^,^^14^

In the chronic phase of CHIKV infection, patients present with musculoskeletal symptoms persisting for more than 3 months.^12^ These are characterized by persistent or recurrent pain, which can be either severe and disabling or milder. The most frequently affected joints are those of the hands, wrists, knees, and ankles, often accompanied by morning stiffness and swelling. About 20% of patients develop neuropathic pain, which is often overlooked and requires specific therapy.^3^

This chronic pain significantly impacts patients' quality of life, hindering daily activities and contributing to emotional disturbances such as depression and sleep disorders. De-Araujo et al. (2019)^12^ investigated the clinical manifestations of chronic musculoskeletal pain after Chikungunya infection. The results revealed that persistent pain, especially in the lower limbs, is a hallmark of the chronic phase, negatively impacting quality of life. Additionally, a reduction in the activation of descending inhibitory pain pathways was observed, suggesting a potential negative interference with endogenous analgesia mechanisms.^12^ These findings underscore the need for multidisciplinary and personalized therapeutic approaches to improve the well-being of such patients.

During this phase, imaging tests, such as radiography and musculoskeletal ultrasound, may be requested to assess joint damage and inflammation.^14^ Patients should undergo serological tests for CHIKV and specific tests to differentiate from other rheumatological diseases. Pharmacological treatment may also include antimalarial drugs, such as hydroxychloroquine and methotrexate, for those with more severe clinical presentations.^11^^,^^14^ Physical therapy and other complementary therapies, such as acupuncture and physical activity, have been recommended alongside pharmacological treatment.^3^^,^^14^

Furthermore, a systematic review conducted by Sales et al. (2023)^15^ evaluated complementary techniques for musculoskeletal rehabilitation. While these approaches show potential for relieving pain, improving muscle strength, increasing the range of motion, and enhancing functional capacity, the studies included in the review exhibited variable methodological quality. Among the interventions with the best evidence, kinesiotherapy, with or without electrothermophototherapy, proved to be effective in reducing pain and improving quality of life, supported by moderate to high-quality evidence.

## Conclusion

Post-arboviral painful chronic musculoskeletal sequelae pose a significant challenge for accurate diagnoses and management. The transition to the chronic phase of these infections may lead to debilitating symptoms, such as persistent pain, severely impacting patients' quality of life. The complexity of the underlying pathophysiological mechanisms, and the variability in clinical presentation, underscore the necessity for pharmacological treatments and multidisciplinary therapeutic approaches.

## Conflicts of interest

The authors declare no conflicts of interest.
